# Comparative analysis of macular characteristics in mCNV and contralateral eyes

**DOI:** 10.3389/fmed.2024.1344968

**Published:** 2024-07-22

**Authors:** Gongyu Huang, Xiangjun She, Yun Zhang, Zongduan Zhang, Lijun Shen

**Affiliations:** ^1^National Clinical Research Center for Ocular Diseases, Eye Hospital, Wenzhou Medical University, Wenzhou, China; ^2^Zhejiang Provincial People’s Hospital (Affiliated People’s Hospital, Hangzhou Medical College), Hangzhou, China

**Keywords:** pathological myopia, choroidal neovasclurization, perforating scleral vessels, myopic macular maculopathies, diffuse choroid atrophy

## Abstract

**Purpose:**

To illustrate the characteristics of perforating scleral vessels in macular regions between mCNV eyes and contralateral eyes in unilateral mCNV patients.

**Methods:**

This was a retrospective study that included patients with unilateral naive mCNV. The study aimed to identify and analyze the distribution of perforating scleral vessels (PSVs) in the macular region of mCNV eyes and contralateral eyes. The central macular choroidal thicknesses (mChT) were measured using optical coherence tomography angiography (OCTA). The grades of myopic atrophic maculopathy (MAM) and macular myopic diffuse chorioretinal atrophy (DCA) were evaluated within groups. The number of PSVs and mChT were compared between contralateral and mCNV eyes based on the grade of DCA. The ROC curves were utilized to explore the diagnostic indexes for mCNV.

**Results:**

A total of 102 eyes from 51 patients with unilateral mCNV were included. There was no significance in the severity of MAM or the grade of DCA between mCNV eyes and contralateral eyes (*p* = 0.074, *p* = 0.054, respectively). The mean number of PSVs in mCNV eyes was fewer than the contralateral eyes [1.00 (1.00–2.00) vs. 2.00 (0.75–3.00), *p* = 0.030]. The mChT in mCNV eyes was thinner than the contralateral eyes [36.00 (25.00–53.75) μm vs. 46.00 (31.00–75.25) μm, *p = 0.001*]. The mean grade of DCA in mCNV eyes was higher than that in contralateral eyes [3.00 (3.00–3.00) vs. 3.00 (2.00–3.00), *p = 0.004*]. When DCA involved the macular region, there were more PSVs in contralateral eyes than in mCNV eyes [1.50 (1.00–2.00) vs. 2.00 (1.00–3.00), *p = 0.042*]. Similarly, when DCA involved the foveal region, there were more PSVs in contralateral eyes than in mCNV eyes [1.50 (1.00–2.00) vs. 3.00 (2.00–4.00), *p* = 0.004]. The grade of DCA and mChT were valuable factors for predicting mCNV eyes (*AUC* = 0.6566, *p* = 0.021; *AUC* = 0.6304, *p* = 0.029; respectively). When the extent of DCA exceeded the foveal region, the count of PSVs was a good diagnostic factor for predicting mCNV (*AUC* = 0.7430, *p* = 0.003).

**Conclusion:**

The mean amount of PSVs was significantly lower in the mCNV eyes compared to the contralateral eyes. When the extent of DCA exceeded the foveal region, the count of PSVs was a good diagnostic factor for predicting mCNV. Myopic eyes with a higher grade of DCA and a thinner mChT were more likely to develop mCNV.

## Introduction

Pathologic myopia is defined as myopia accompanied by typical degenerative lesions, including myopic maculopathies that are equal to or more severe than diffuse chorioretinal atrophy (DCA). DCA is a grade 2 lesion in the META-analysis classification system for pathologic myopia (1). The appearance of a posterior staphyloma is also considered a typical degenerative lesion in pathologic myopia (2). Pathologic myopia is a significant cause of visual impairment and blindness in East Asia, with an estimated prevalence of 1 to 3% in the population. The accompanying degenerative changes in the posterior segment of the eye and the macula can lead to vision loss, and pathologic myopia is one of the main causes of blindness in East Asia (3).

Myopic choroidal neovascularization (mCNV) has been estimated to develop in 5 to 10% of eyes with pathological myopia, and it is the most common cause of vision loss among all myopic-related macular complications (4, 5). The prognosis of mCNV without clinical treatment is poor (6–8). A decade-long study showed that vision decreased significantly to 20/200 or less in untreated mCNV patients followed for 10 years (7).

Cohen SY et al. investigated the population with mCNV and showed that about 14% of patients were diagnosed with bilateral naive mCNV, which means that most patients suffered from unilateral mCNV (9). About 15% of patients diagnosed with naive mCNV in unilateral eyes developed mCNV in the contralateral eyes within 8 years of follow-up (5, 6).

Lacquer cracks and patchy atrophies have been hypothesized to play a role in the pathogenesis of mCNV in patients with pathological myopia, and the apparent thinning of the choroid and loss of choroidal vessels in pathologic myopia may lead to the progression of myopic macular atrophy (10, 11). Monitoring for signs of mCNV in patients with pathological myopia, and early diagnosis and treatment of mCNV, can help preserve vision and prevent further vision loss.

However, Querques et al. reported that perforating scleral vessels (PSVs) were usually detected in lacquer cracks (12). Ruiz-Medrano et al. reported that PSVs were detected in more than 90% of mCNV eyes (13). They speculated that PSV might contribute to the formation of myopic maculopathy. In our previous study, we found that the eyes with PSVs adjacent to mCNV had worse efficacy of anti-VEGF treatment (14).

Whether the structure of PSV was an important sign for mCNV was not known. This study aimed to analyze the characteristics of PSVs in the macular region between mCNV eyes and contralateral eyes in patients with unilateral mCNV. Additionally, we aimed to identify risk factors for mCNV within the macular region.

### Methods

Ethics approval for this retrospective observational study was obtained from the Research Ethics Committee of the Affiliated Eye Hospital of Wenzhou Medical University, China. The ethics acceptance number is H2023-012-K-09, and the registration number of this clinical trial is ChiCTR2300070120. All procedures for this study were conducted in accordance with the Declaration of Helsinki. We reviewed the medical records of patients who visited the Affiliated Eye Hospital of Wenzhou Medical University from November 2017 to September 2022.

The inclusion criteria for this study were as follows: (1) Patients with bilateral eyes met the following diagnostic criteria for high myopia: spherical refraction ≤ −6.00D or axial length longer than 26 mm, with typically degenerative changes in the retina, choroid, and sclera; (2) presence of naive mCNV: ① appeared as a flat, small, greyish subretinal lesion beneath or in close proximity to the fovea with or without hemorrhage on fundus photography; ② assessed on en face images generated by the automatically segmented outer retina slabs and choriocapillaris slabs, mCNV appeared as a large hyperintense vascular anastomotic network on OCT and OCTA devices; ③ B-scan images on OCT/OCTA appeared as highly reflective dome-shaped elevations above the retinal pigment epithelial band (because most mCNVs were type 2 CNVs), ④ FA findings in mCNV usually comprise well-defined hyper fluorescence in the early phase with leakage in the late phase in a classic CNV pattern of leakage (15, 16). The exclusion criteria were as follows: (1) CNV was secondary to other diseases, such as multifocal choroiditis, panuveitis, idiopathic CNV, and neovascular AMD; (2) the imaging quality of OCTA and fundus images was poor, or OCTA images showed poor correspondence in the macular area; (3) coexisting or history of any other severe ocular or systemic disease, or undergone other intraocular surgeries in addition to cataract or refractive surgery.

The age, gender, best corrected visual acuity (BCVA) measured with a Landolt C chart, and spherical equivalent (SE) were collected from the medical records. The grade of myopic chorioretinal atrophy (MAM) was determined by color fundus photography (TRC-50DX, Topcon Corporation, Tokyo, Japan) and confocal scanning laser ophthalmoscopy (cSLO; Optos Daytona, Optos, England). SD-OCT (Spectralis SD-OCT; Heidelberg Engineering, Germany) was used to measure the central macular choroidal thickness (mChT). Horizontal and vertical B-scans, as well as en-face images, mainly included outer retina slabs, choriocapillaris slabs, and choroid slabs automatically generated within the 3 × 3 mm, 6 × 6 mm, and 9 × 9 mm by OCTA (Angio OCT; Optovue, Fremont, CA, United States) and swept-source OCTA (SS-OCTA) (VG200D; SVision Imaging, Ltd., Luoyang, China) were used to assess the number and distribution of PSVs. Fluorescein angiography was performed when necessary.

### Acquisition and analysis of PSVs’ distribution

**Figure 1:**
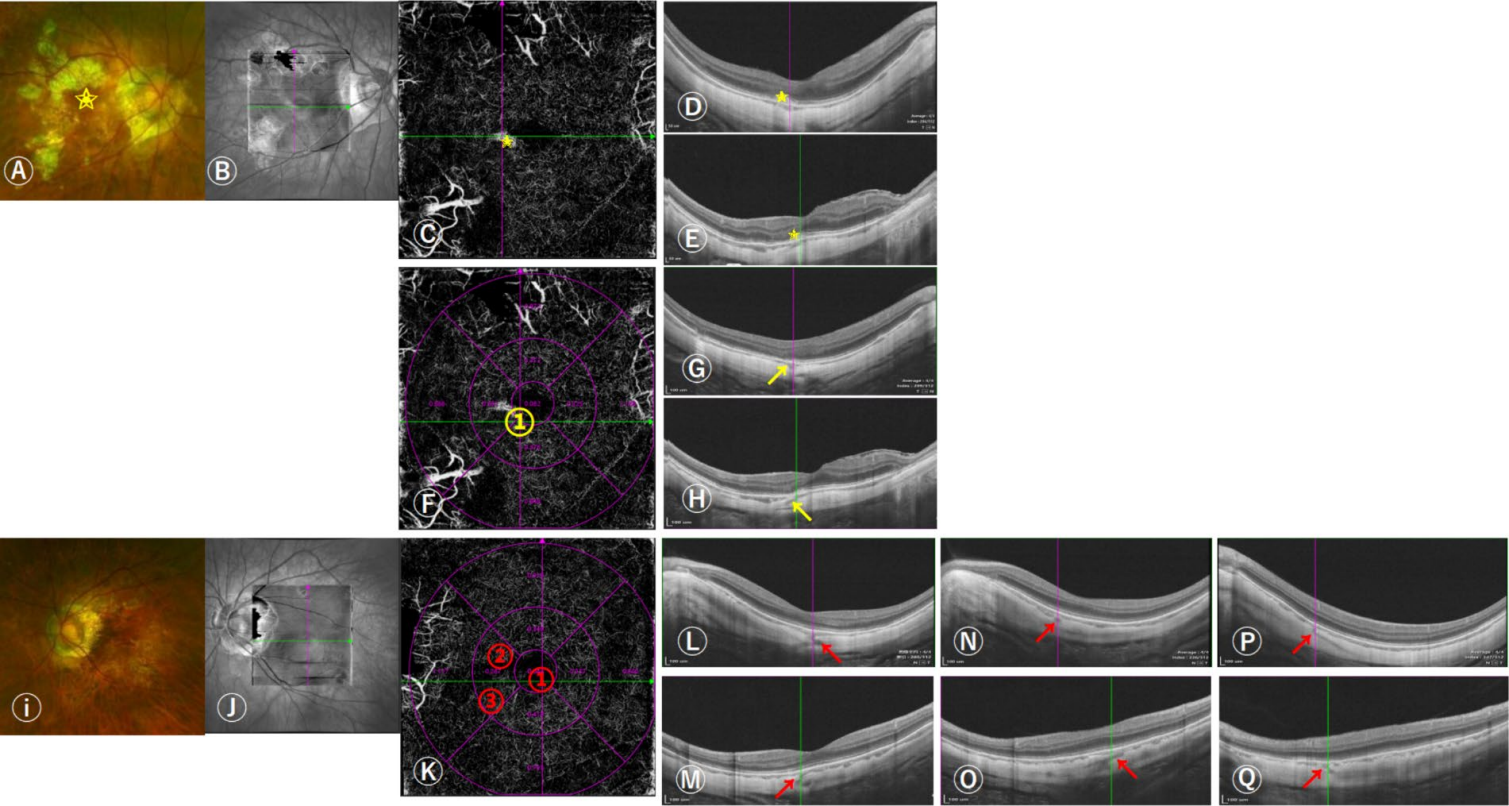
Images of the distributions of perforating scleral vessels (PSVs) in the macular region using swept-source OCT (SS-OCT). **(A)** 57-year-old woman was diagnosed with unilateral naive myopic choroidal neovascularization (mCNV) in her right eye; her equivalent refractive error of OD was −11.75 D and OS was −10.38 D. She had one PSV in her right eye and three in her left eye. The scanning laser ophthalmoscopy and OCT images indicated that OD was graded as A3T0N2s, OS was graded as A2T0N0, and the grade of DCA was D3. The boxes **(B,J)** indicated the scan area on SS-OCT. **(A–H)** The right fundus shows a grayish mCNV (yellow star in **A,C–E**) and **(D,E)** the corresponding horizontal and vertical B-scan images of mCNV. **(F)** The cross lines indicate the location of the PSV and are marked with a yellow number (yellow arrows in **G,H**) indicating horizontal and vertical B-scan images corresponding to the location of the PSV in figure **(F)**. **(I–Q)** Left fundus without mCNV, **(K)** the cross lines indicated the location of the first PSV, and the locations of the three PSVs entering the choroid are marked with red numbers. **(L,M)** indicated horizontal and vertical B-scan images corresponding to the location of the first PSV, **(N,O)** the location of the second PSV, and **(P,Q)** the location of the third PSV.

The criteria for defining PSVs were as follows: (1) linear or wavy morphology in OCTA images; (2) low reflectance appearance; (3) extension from the sclera through the choroid toward the retina (12, 17). The position of PSVs was determined by where they joined the choroid based on en-face images manually adjusted the thickness of contiguous slabs of choroid and sclera on OCTA combined with horizontal and vertical B-scans. When necessary, fluorescein angiography results were also used to confirm the position of PSVs (Figure 1).

The ETDRS grid, which was automatically generated on the OCTA device, was used to determine the relative position between the PSVs and the fovea within the macular region (3 × 3 mm area with the fovea as the center). The innermost ring of the ETDRS grid was 1 mm in diameter, and the middle ring was 3 mm in diameter. The ETDRS grid was positioned manually to correspond best to the position of the center of the fovea. Meanwhile, 6 × 6 mm and 9 × 9 mm scans on OCTA were used to check the accuracy of the distribution of PSVs at the edge of the 3 × 3 mm scan area. The total number of PSVs was recorded by counting all PSVs within the macular region. In addition, we divided the position of PSVs into five types: (1) PSVs in the foveal area; (2) in the superior area of the fovea; (3) in the inferior area of the fovea; (4) in the nasal area of the fovea; (5) in the temporal area of the fovea. (Figure 1) Two experienced retinal doctors repeated the measurements three times to confirm the presence of PSVs, the disagreement was decided by a senior expert. The distributions and counts of PSVs in both eyes were compared in patients with unilateral as well as bilateral mCNV.

### Definition and grading of myopic maculopathy (MM)

Fundus photographs combined with B-scan images on OCTA were analyzed, and the macular microvasculature was also assessed. According to a recent international classification system (ATN), myopic maculopathies are divided into three types: myopic atrophic maculopathy (MAM), myopic tractional maculopathy (MTM), and myopic neovascularization (MNM) (1). The MAM is further classified into 5 grades: A0 (normal macula without any myopic retinal lesions), A1 (tessellated fundus only), A2 (diffuse chorioretinal atrophy), A3 (patchy chorioretinal atrophy), and A4 (complete macular atrophy). Diffuse chorioretinal atrophy (DCA) is a common pathological change in highly myopic eyes.

DCA appears as a faint yellowish lesion in color fundus photography and usually starts from the temporal side of the peripapillary region and gradually involves the entire posterior pole (18). The central macular choroidal thickness in eyes with diffuse chorioretinal atrophy (usually less than 100 μm) is thinner than that in eyes with a tessellated fundus, and the choroidal capillary density is significantly reduced. This disproportionate thinning of the choroid may be a key factor in diffuse atrophy and pathologic myopia (19). The distance from the fovea to the temporal edge of the optic disc was denoted as 3 mm, half of which was equal to the radius of the middle circle of the Early Treatment Diabetic Retinopathy Study (ETDRS) grid and one-sixth of which was equal to the radius of the inner circle of the grid. In these eyes with a diagnosis of DCA, we subclassified diffuse chorioretinal atrophy (DCA) according to the extent of atrophic changes on fundus photographs into 4 grades: without DCA or with DCA but without involvement of a radius of 3 mm around the fovea (D0); involvement to a radius of 1.5 to 3 mm around the fovea (D1); involvement of the parafovea (D2); and involvement of the fovea (D3) (20). MTM were classified into 6 grades: T0 (no macular schisis), T1 (inner or outer foveoschisis), T2 (inner and outer foveoschisis), T3 (foveal retinal detachment), T4 (full-thickness macular hole), and T5 (T4 + retinal detachment). The central macular choroidal thickness (mChT) was measured three times on B-scan images using SD-OCT.

### Statistical analysis

The Kolmogorov–Smirnov test was used to check the normality of numerical variables. Normally distributed variables were presented as mean ± SD, non-normally distributed variables were presented as median (the first quartile to the third quartile), and *P* < 0.05 was considered statistically significant and shown in bold in the tables. The independent t-test, or Mann–Whitney *U* test, was used to compare continuous variables. Non-continuous variables were compared based on the Mann–Whitney *U* test. The Wilcoxon signed-rank test was used to compare the count of PSVs between bilateral eyes. Receiver operating characteristic (ROC) curves were plotted to assess the ability of PSVs and mChT to distinguish a subject with mCNV. The area under the ROC curve (AUC) was used to determine the diagnostic accuracy of the indexes mentioned above. The closer the value of the AUC was to 1.0, the more perfect the discrimination was (21). All data were analyzed using the commercial analytical software program SPSS 26.0.0 (SPSS Inc., Chicago, IL). GraphPad Prism 8.3.0 (GraphPad Prism Inc., San Diego, California, United States) was used to calculate relevant metrics and plot the ROC curves.

## Results

### Demographics and fundus characteristics of participants

102 eyes of 51 patients (11 males and 40 females) diagnosed with unilateral naive mCNV at their initial visit were enrolled. (Table 1) In patients with unilateral mCNV, the mean age was 53.51 ± 14.01 years old, the mean spherical equivalent (SE) was −13.03 ± 3.12D, the mean SE of contralateral eyes was −12.43 ± 4.71D (*p* = 0.074). The mean BCVA of mCNV eyes was 0.40 (0.22–0.85), and that of contralateral eyes was 0.22 (0.05–0.40) (*p* = 0.000).

**Table 1:** Demographic profiles and fundus characteristics of participants with unilateral mCNV.

	CNV eye (*n* = 51)	Contralateral eye (*n* = 51)	*p* value
Age, *y*	53.51 ± 14.01		
Male, *n* (%)	11 (22%)		
SE,D	−13.03 ± 3.12	−12.43 ± 4.71	0.074^*^
BCVA, logMAR	0.40 (0.22–0.85)	0.22 (0.05–0.40)	0.000^†^
MAM			
Grade, *n* (%)	2.00 (2.00–3.00)	2.00 (2.00–2.00)	0.054^‡^
0	0	0	
1	1 (2%)	2 (4%)	
2	33 (65%)	42 (82%)	
3	13 (25%)	2 (4%)	
4	4 (8%)	5 (10%)	
DCA			
Number (%)	33 (66%)	42 (82%)	
Grade, *n* (%)	3.00 (3.00–3.00)	3.00 (2.00–3.00)	0.004^†^
0	0	1 (2%)	
1	1 (3%)	6 (14%)	
2	4 (12%)	12 (29%)	
3	28 (85%)	23 (55%)	
MTM	0.00 (0.00–1.00)	0.00 (0.00–0.00)	0.171^†^
mChT, μm	36.00 (25.00–53.75)	46.00 (31.00–75.25)	0.001^‡^
PSV count			
Total	1.00 (1.00–2.00)	2.00 (0.75–3.00)	0.030^‡^
Foveal	0.00 (0.00–0.25)	0.00 (0.00–0.00)	0.971^†^
Parafovea	1.00 (0.00–2.00)	2.00 (0.00–3.00)	0.028^‡^
Superior	0.00 (0.00–1.00)	0.00 (0.00–1.00)	0.183^†^
Inferior	0.00 (0.00–0.00)	0.00 (0.00–1.00)	0.011^‡^
Nasal	0.00 (0.00–1.00)	0.00 (0.00–1.00)	0.618^†^
Temporal	0.00 (0.00–0.00)	0.00 (0.00–1.00)	0.479^†^

BCVA, best corrected visual acuity; MAM, myopic macular atrophy.

DCA, diffuse chorioretinal atrophy; MTM, myopic macular traction.

mChT, central macular choroidal thickness; PSV, perforating scleral vessels.^*^Student’s *t*-test. ^†^Mann–whitney *U* test. ^‡^Wilcoxon signed-rank test.

There was no significant difference in the grade of MAM and MTM between mCNV eyes and contralateral eyes (*p* = 0.054, *p* = 0.171, respectively). In the MAM classification analysis, the proportion of eyes with DCA was highest in both mCNV eyes (66%) and contralateral eyes (82%). In mCNV eyes, 0 (0%) eyes were categorized as D0, 1 (3%) eye as D1, 4 (12%) eyes as D2, and 28 (85%) eyes as D3. In contralateral eyes, 1 (2%) eye was categorized as D0, 6 (14%) eyes as D1, 12 (29%) eyes as D2, and 23 (55%) eyes as D3. The mean grade of DCA in mCNV eyes was higher than in contralateral eyes (3.00 (3.00–3.00) vs. 3.00 (2.00–3.00), *p* = 0.004).

The average central macular choroidal thickness (mChT) of mCNV eyes was thinner than that of contralateral eyes: 36.00 (25.00–53.75) μm vs. 46.00 (31.00–75.25) μm (*p* = 0.029). Regarding the count and distribution of PSVs within the macular region, there were more PSVs in contralateral eyes than in mCNV eyes (*p* = 0.030), especially in the parafoveal and inferior foveal regions (*p* = 0.028) (*p* = 0.011).

### Binocular features of PSVs in eyes with the same grade of DCA

The structures of PSVs were compared in mCNV eyes and contralateral eyes based on the same grade of DCA. When DCA involved the macular region (grade of DCA equal to D2 and D3), the mean BCVA of mCNV eyes was worse than that of the contralateral eyes (*p* = 0.000), and the count of PSVs in the macular region of mCNV eyes was less than that in the contralateral eyes [1.50 (1.00–2.00) vs. 2.00 (1.00–3.00); *p* = 0.042]. Similarly, when DCA involved the fovea (grade of DCA equal to D3), the mean BCVA of mCNV eyes was worse than that of contralateral eyes (*p = 0.000*), and the count of PSVs in the macular region of mCNV eyes was less than that in the contralateral eyes [1.50 (1.00–2.00) vs. 3.00 (2.00–4.00); *p = 0.004*]. However, the number of PSVs in the foveal region of bilateral eyes was not significantly different, regardless of the grade of DCA (*p = 0.947* for D2 + D3; *p = 0.453* for D3) (Table 2).

**Table 2:** The features of PSVs in patients with unilateral mCNV according to the degree of DCA.

	Macula involved (D2 + D3)			Fovea involved (D3)		
	mCNV eye	Contralateral eye		mCNV eye	Contralateral eye	
	(*n* = 32)	(*n* = 35)	*P* value	(*n* = 28)	(*n* = 23)	*P* value
Age,y	53.06 ± 13.49	52.11 ± 14.74	0.605*	53.61 ± 12.71	52.96 ± 15.68	0.310*
SE,D	−12.51 ± 3.11	−11.82 ± 3.44	0.681*	−12.71 ± 3.27	−12.20 ± 3.80	0.399*
BCVA,logMAR	0.46 (0.24–0.96)	0.10 (0.05–0.22)	0.000^†^	0.46 (0.30–0.82)	0.10 (0.05–0.22)	0.000^†^
mChT,μm	38.00 (25.00–54.00)	45.00 (30.75–62.00)	0.158^†^	36.00 (24.25–51.75)	38.00 (25.00–53.00)	0.472^†^
PSV count						
Total	1.50 (1.00–2.00)	2.00 (1.00–3.00)	0.042^†^	1.50 (1.00–2.00)	3.00 (2.00–4.00)	0.004^†^
Foveal	0.00 (0.00–0.75)	0.00 (0.00–1.00)	0.947^†^	0.00 (0.00–0.75)	0.00 (0.00–1.00)	0.453^†^

BCVA, best corrected visual acuity; mChT, central macular choroidal thickness; PSV, perforating scleral vessels. ^*^Student’s *t*-test. ^†^Mann–whitney *U* test.

### The diagnostic value of PSVs for mCNV

**Figure 2:**
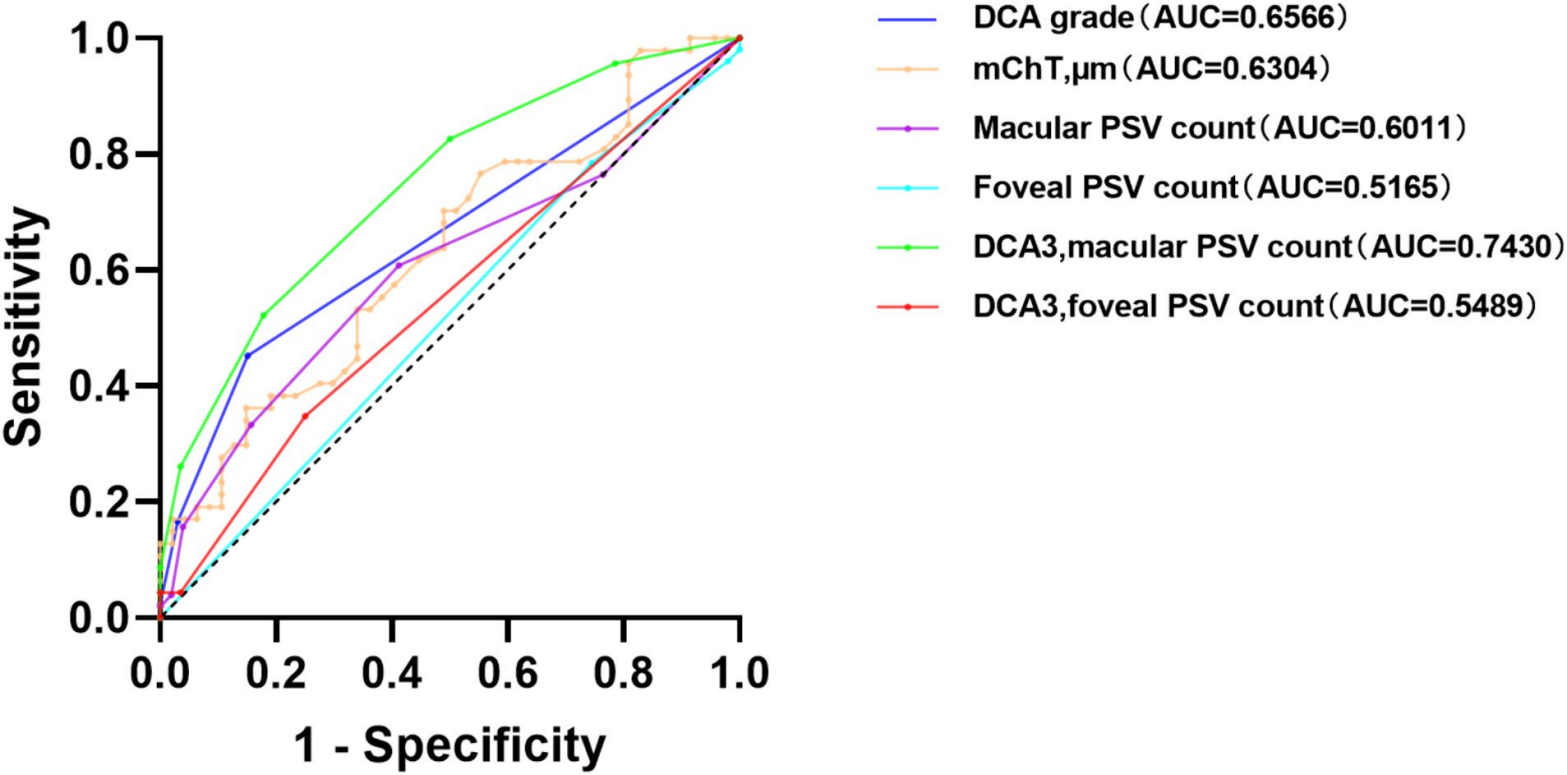
Receiver operating characteristic (ROC) curves of parameters for mCNV.

Receiver operating characteristic (ROC) curves were used to analyze the diagnosis value for mCNV. The results in Figure 2 depict that the grade of DCA showed an Area Under the Curve (AUC) of 0.6566 and a cut-off value of 2.50, producing an 84.9% specificity and 45.2% sensitivity with a *p*-value of 0.021. The mChT produced an AUC of 0.6304 and a cut-off value of 30.50 μm with a 44.7% specificity and 76.6% sensitivity with a *p*-value of 0.029. Most of the eyes in our study showed a grade of DCA equal to D3. To exclude the effects of chorioretinal atrophy, the ROC curve analysis of the number of PSVs in the macular region when the grade of DCA was equal to D3 showed an AUC of 0.7430 and the cut-off value of 2.50, producing an 82.1% specificity and 52.2% sensitivity with a *p*-value of 0.003. Therefore, the grade of DCA, mChT, and the number of PSVs of eyes diagnosed with D3 were all potentially useful diagnostic factors for mCNV. However, further studies are required to confirm the diagnostic value of these factors for mCNV.

The number of PSVs in the macula or foveal region was not a perfect diagnostic factor, with AUC values of 0.6011 (*p* = 0.078) and 0.5165 (*p* = 0.733), respectively. Also, the number of PSVs in the foveal region was not a perfect diagnostic factor when the DCA grade was equal to D3, with an AUC value of 0.5489 and a *p*-value of 0.551 (Table 3).

**Table 3:** Results of important parameters from ROC curve.

Variables	AUC	95% confidence interval	*P* value	Youden’s J statistic	Cut-off value
DCA grade	0.6566	0.54–0.78	0.021	0.3009	2.50
mChT, μm	0.6304	0.52–0.74	0.029	0.2128	30.50
Macular PSV count	0.6011	0.49–0.71	0.078		
Foveal PSV count	0.5165	0.40–0.63	0.773		
DCA = 3, macular PSV count	0.7430	0.61–0.88	0.003	0.3431	2.50
DCA = 3, foveal PSV count	0.5489	0.39–0.71	0.551		

DCA, diffuse chorioretinal atrophy; mChT, central macular choroidal thickness; PSV, perforating scleral vessels; AUC, Area under the curve.

## Discussion

This study investigated the differences in the mChT and the number of PSVs in the macular region in patients with unilateral mCNV. The results showed that mChT was thinner and there were fewer PSVs in the macular region in mCNV eyes compared to the contralateral eyes. When the extents of DCA were closer to the fovea, there were more PSVs in the macular region in the contralateral eyes than in the mCNV eyes. When DCA approached the fovea, the number of PSVs in the macular region might be a potentially useful diagnostic factor for mCNV.

Previous research suggests that mCNV may be correlated with myopic atrophic maculopathy (MAM) (22–24). Some researchers reported that there was no significant correlation between mCNV and myopic tractional maculopathy (MTM) (25). One study by Flores-Moreno I et al. showed that in myopic eyes, the thickness of the choroid decreased (26). In our study, we did not find any significant difference in SE, grade of MAM, or MTM between the bilateral eyes of either unilateral or bilateral mCNV patients. However, our results suggest that the mChT was thinner in the mCNV eyes than in the contralateral eyes. This finding supports the theory that mCNV may be associated with choroidal thinning, which could be a potential risk factor for mCNV. Further studies are necessary to confirm this association and investigate the underlying mechanisms involved.

Ishida T et al. reported a connection between mCNV and intrascleral vessels. They found that 75% of PSVs were detected below or around the mCNV, and indocyanine green angiography showed that these PSVs were intrascleral arteries originating from the short posterior ciliary arteries (SPCAs). Swept-source OCT showed that some of the mCNVs had continuous connections with scleral vessels, mainly the SPCA (27). PSVs are commonly observed in highly myopic eyes. Some studies also suggest that PSVs may originate from the posterior ciliary artery (PCA) and SPCA (28, 29). This suggests that PSVs merge into the choroid and provide blood flow and oxygen diffusion to the choroid.

Asakuma et al. (30) reported a high prevalence of DCA (91.6%) in patients with myopic retinopathy. However, Liu et al. (20) reported a lower prevalence of DCA (20.6%) in the high myopic group. Our study showed that a large proportion of both mCNV eyes and contralateral eyes had DCA, with proportions of 66 and 82%, respectively. The average grade of DCA in mCNV eyes was also higher than in contralateral eyes, suggesting that the extent of DCA was closer to the fovea in mCNV eyes.

In our study, we observed more PSVs in contralateral eyes than mCNV eyes in the macular region, especially in the parafovea area of the retina and the inferior quadrant of the fovea. Also, when the DCA involved the macula, we found more PSVs in the contralateral eyes than in the mCNV eyes. This finding is interesting because it suggests that in highly myopic eyes where the choroid is almost non-existent, in eyes with slightly more PSV counts, the probability of mCNV being detected may be smaller. There may be a negative correlation between PSV and mCNV, but the role of PSV in the generation of mCNV and the mechanism of this are not yet clear. We speculate that in highly myopic eyes with an almost absent choroid, when the blood flow from PSVs is weakened, mCNVs are more likely to appear in the macular area. These findings suggest that in highly myopic eyes, the choroidal blood flow may be insufficient to nourish the retinal tissue, leading to the development of mCNV.

By analyzing AUC values under ROC curves, we found that the grade of DCA >2.5 and the mChT <30.5 μm were the diagnostic factors for mCNV. When the grade of DCA was D3, the number of PSVs in the macular region was less than 2.5, which was also an effective diagnostic factor for mCNV. These results suggest that in highly myopic eyes accompanied by atrophied choroids, PSVs may play an important role in the blood flow supply for choroids. When there are fewer PSVs in the macular region, this phenomenon may be weakened, and the probability of the development of mCNV may increase.

We acknowledge that our study had several limitations. First, the sample size was relatively small, and a larger sample size experiment is needed in the future to verify our current research results. Second, this was a retrospective cross-sectional clinical study that had limited research capacity on the role of PSVs in the pathogenesis of mCNV and required appropriate animal model experiments for verification. Third, histopathological analysis was needed to verify the connection between mCNV and intrascleral vessels, and more relevant studies are needed to explore the pathological mechanism in the future.

## Data availability statement

The raw data supporting the conclusions of this article will be made available by the authors, without undue reservation.

## Ethics statement

The studies involving humans were approved by the Research Ethics Committee of the Affiliated Eye Hospital of Wenzhou Medical University. The studies were conducted in accordance with the local legislation and institutional requirements. Written informed consent for participation was not required from the participants or the participants’ legal guardians/next of kin in accordance with the national legislation and institutional requirements.

## Author contributions

GH: Data curation, Investigation, Writing – original draft. XS: Data curation, Funding acquisition, Methodology, Writing – review & editing. YZ: Project administration, Supervision, Writing – review & editing. ZZ: Conceptualization, Supervision, Validation, Visualization, Writing – review & editing. LS: Conceptualization, Supervision, Validation, Visualization, Writing – review & editing.
